# Congenital Diverticular Disease of the Entire Colon

**DOI:** 10.1155/2013/319026

**Published:** 2013-04-11

**Authors:** A. Patel, H. M. N. Joshi, C. Kaur, J. M. Wilson

**Affiliations:** ^1^Department of General Surgery, Whittington Hospital, Magdala Avenue, Archway, London N19 5NF, UK; ^2^Department of Histopathology, Whittington Hospital, Magdala Avenue, Archway, London N19 5NF, UK

## Abstract

Congenital or true colonic diverticulosis is a rare condition typified by the preservation of the colonic wall architecture within the diverticular outpouching. Cases of multiple jejunal diverticula have been reported as well as cases of solitary giant diverticula of the colon. There have been no reports in the literature of pancolonic congenital diverticulosis.

## 1. Introduction

Congenital or true colonic diverticulosis is a rare condition typified by preservation of the colonic wall architecture within the diverticular outpouching. Cases of multiple jejunal diverticula have been reported and are probably secondary to points of focal weakness from the insertion points of vessels into the wall of the colon [1]. Other cases of solitary giant diverticula of the colon have been reported [2]. Of these, 13% were identified as true diverticula [3]. There have been no reports in the literature of pancolonic congenital diverticulosis. 

## 2. Case Presentation

A 26-year-old man of Bangladeshi origin presented with a two-day history of generalised abdominal pain, loss of appetite, and nausea. His past medical history includes hypertension, type 1 diabetes mellitus, and autosomal dominant polycystic kidney disease. He is the product of a consanguineous marriage and suffered from intrauterine growth retardation and failure to thrive with persistent short stature and microcephaly. Previous investigations for chromosomal disorders at Great Ormond Street Hospital were negative, and the karyotype was confirmed as 46XY.

On examination, he was afebrile and physiologically stable with tenderness in the lower abdomen and a BMI of 14 (height 135.5 cm and weight 25.56 kg). Inflammatory markers were mildly raised (white cell count 13.2, neutrophils 8.4, and C-reactive protein 87). He was admitted for observation, intravenous fluids and antibiotics. A computed tomography scan of the abdomen and pelvis revealed mild thickening of the descending colon but no focal inflammatory changes, free air, or free fluid.

The patient deteriorated clinically and underwent a diagnostic laparoscopy, which identified a thickened inflamed loop bowel adherent to the anterior abdominal wall. A laparotomy was performed. There was extensive diverticular disease and an associated inflammation of the entire colon with an inflammatory mass around the transverse colon adherent to the anterior abdominal wall. The diverticula were large and broad based, resembling jejunal diverticulosis macroscopically. Malignancy could not be excluded, and the tissues were extremely friable, so a subtotal colectomy and end ileostomy with abdominal washout were performed. Our patient's postoperative course was complicated by an episode of aspiration pneumonia from which he made a full recovery. 

**Figure 1:**
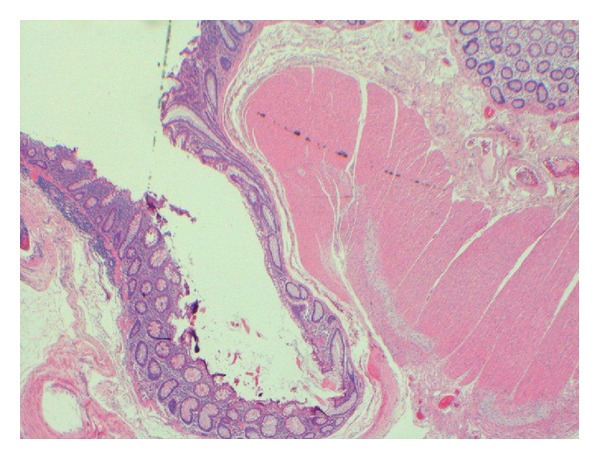
Section of caecal diverticulum showing the outpouching of mucosa and preservation of the muscularis propria excludeing pseudodiverticulum.

Histopathological examination supported the diagnosis of true pancolonic diverticulosis. The entire specimen showed numerous large diverticula and associated inflammation/abscess formation. The majority of the diverticula had a colonic type muscularis propria and can therefore be considered as true diverticula resulting from congenital malformation (Figure 1). Lymph nodes showed reactive changes only. Subsequent elastin immunohistochemical staining was within normal limits.

He was discharged 13 days postoperatively, with a view to restoration of intestinal continuity in 6 months.

## 3. Discussion

Although uncommon, isolated congenital diverticula of the colon have been described in only a small number of case reports [3], but not in the context of a generalised diverticulosis involving the whole colon. The acquired diverticula of the colon are pseudodiverticula involving herniation of colonic mucosa through the bowel wall. This occurs at sites of perforation of the bowel wall by the vasa recta typically on the antimesenteric taenia coli. Although the exact mechanism remains unclear, their development is thought to be related to intestinal dyskinesia and raised intraluminal pressures [4]. In contrast, true diverticula involve all layers of the bowel wall and are predominately solitary and right sided. To our knowledge, this is the first reported case of congenital diverticulosis affecting the entire colon; therefore, there is little within the literature describing potential aetiologies. The patient's previous medical history suggests that the aetiology could be related to an undiagnosed congenital syndrome or connective tissue disorder/variant. Diverticular disease may be an extrarenal manifestation of polycystic kidney disease [5].

## 4. Conclusion

We report the first documented case of generalised congenital colonic diverticulosis affecting the entire colon resulting in an acute presentation.
